# 1,8-cineole, a TRPM8 agonist, is a novel natural antagonist of human TRPA1

**DOI:** 10.1186/1744-8069-8-86

**Published:** 2012-11-29

**Authors:** Masayuki Takaishi, Fumitaka Fujita, Kunitoshi Uchida, Satoshi Yamamoto, Maki Sawada (Shimizu), Chihiro Hatai (Uotsu), Mayumi Shimizu, Makoto Tominaga

**Affiliations:** 1 Division of Cell Signaling, Okazaki Institute for Integrative Bioscience (National Institute for Physiological Sciences), National Institutes of Natural Sciences, Okazaki, 444-8787, Japan; 2 Central Research Laboratories, Mandom Corporation, Osaka, 540-8530, Japan; 3 Department of Physiological Sciences, The Graduate University for Advanced Studies, Okazaki, 444-8585, Japan

**Keywords:** 1,8-cineole, Pain relief, TRP channels, TRPA1

## Abstract

**Background:**

Essential oils are often used in alternative medicine as analgesic and anti-inflammatory remedies. However, the specific compounds that confer the effects of essential oils and the molecular mechanisms are largely unknown. TRPM8 is a thermosensitive receptor that detects cool temperatures and menthol whereas TRPA1 is a sensor of noxious cold. Ideally, an effective analgesic compound would activate TRPM8 and inhibit TRPA1.

**Results:**

We screened essential oils and fragrance chemicals showing a high ratio of human TRPM8-activating ability versus human TRPA1-activating ability using a Ca^2+^-imaging method, and identified 1,8-cineole in eucalyptus oil as particularly effective. Patch-clamp experiments confirmed that 1,8-cineole evoked inward currents in HEK293T cells expressing human TRPM8, but not human TRPA1. In addition, 1,8-cineole inhibited human TRPA1 currents activated by allyl isothiocyanate, menthol, fulfenamic acid or octanol in a dose-dependent manner. Furthermore, *in vivo* sensory irritation tests showed that 1,8-cineole conferred an analgesic effect on sensory irritation produced by TRPA1 agonists octanol and menthol. Surprisingly, 1,4-cineole, which is structurally similar and also present in eucalyptus oil, activated both human TRPM8 and human TRPA1.

**Conclusions:**

1,8-cineole is a rare natural antagonist of human TRPA1 that has analgesic and anti-inflammatory effects possibly due to its inhibition of TRPA1.

## Background

Essential oils are often used in alternative medicine as analgesic and anti-inflammatory remedies. However, the specific compounds that confer the effects of essential oils and the molecular mechanisms are largely unknown. For example, linalool, a monoterpene compound commonly found as a major component of several essential oils has been reported to produce antinociception in two different pain models in mice although the mechanism of its analgesic effects is unknown [1].

Transient receptor potential (TRP) channels respond to a wide variety of sensory stimuli, including temperature, nociceptive compounds, touch, osmolarity, and pheromones [2-4]. TRPA1 is a TRP channel that functions as a receptor for noxious cold temperatures and allyl isothiocyanate (AITC), the pungent ingredient of mustard oil [5-9]. Although the role of TRPA1 in sensing noxious cold and somatic mechanosensation *in vivo* remains unsettled, especially in mammals [6,7,10], TRPA1 is an established chemical nocisensor for a wide variety of reactive compounds. TRPA1 is a receptor for the irritation induced by parabens on the skin [11] and for pain produced by alkaline pH [12]. TRPA1 is also activated by flufenamic acid (FFA), 2-aminoethoxydiphenyl borate (2-APB), icilin, menthol, intracellular calcium or zinc ions [8,13-21]. However, menthol has different effects on TRPA1 in human and mouse. A previous study identified a bimodal action of mouse TRPA1 (mTRPA1) gating by menthol: submicromolar to low micromolar-concentrations of menthol cause robust channel activation, whereas higher concentrations lead to a reversible channel block. Such bimodal action is not observed on human TRPA1 (hTRPA1) [22,23]. TRPA1 has also been reported to be involved in inflammation produced by several airway irritants that cause asthma [24,25]. TRPA1 is an excitatory ion channel targeted by cold nociception and inflammatory pain. Therefore, TRPA1 is considered to be a promising target for use in identifying analgesic drugs [26-31]. Moreover, TRPM8 is a thermosensitive receptor that detects cool temperatures and menthol [32,33], a natural non-reactive cooling compound, which is also involved in antinociception to some extent [34,35]. Menthol, the main ingredient of peppermint, is used for pain relief in daily life through TRPM8 activation [35,36]. However, high doses of menthol caused sensory irritation [37] because it acts as a TRPA1 activator in humans [23]. Camphor, another essential oil component, is now known to exert analgesic effects probably through inhibition of TRPA1 [31] and activation of TRPM8 [38]. However, camphor is not suited for use as an analgesic compound because it causes a warm and hot sensation [39], probably through TRPV1 activation [31]. Therefore, we thought an effective analgesic compound would activate TRPM8 and inhibit TRPA1, but not activate TRPV1.

Several TRP channels are known to be activated or inhibited by plant-derived substances, such as menthol and camphor, some of which are contained in essential oils. Essential oils have been used for a long time and their side effects are generally considered to be minimal. Accordingly, essential oils, especially ones acting on TRP channels, could be a promising source for the development of analgesic agents.

Therefore, we have been screening essential oils for the ability to activate human TRPM8 (hTRPM8) but not hTRPA1, distinct from menthol. Through the screening, we found that eucalyptus oil exhibited relatively high hTRPM8-activating ability with less activation of hTRPA1. Furthermore, 1,8-cineole, a main component of eucalyptus oil, was identified as a novel natural antagonist of hTRPA1.

## Results

### Eucalyptus oil shows hTRPM8-activating ability with little activation of hTRPA1

**Figure 1:**
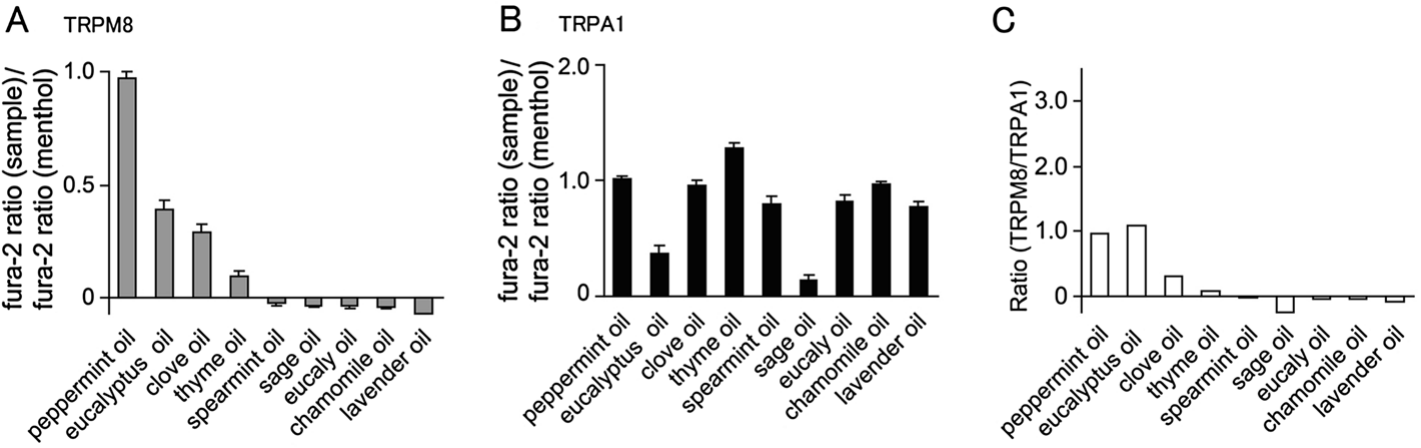
**Abilities of essential oils to activate hTRPM8 or hTRPA1. (A, B)** Comparison of the effects of essential oils (0.01 wt%) on hTRPM8 (n = 34–134) (**A**) or hTRPA1 (n = 16–67) (**B**) using a Ca^2+^-imaging method with HEK293T cells expressing hTRPM8 or hTRPA1. Fura-2 ratio (340/380 nm; cytosolic Ca^2+^ concentrations) increases by each oil were normalized to the fura-2 ratio increases by 1 mM menthol. (**C**) The Ratio of hTRPM8-activating ability versus hTRPA1-activating ability by dividing the values in (**A**) by the values in (**B**).

First, in order to find promising essential oils for development of analgesics, we evaluated the effects of essential oils (0.01 wt%) by comparing their abilities to activate hTRPM8 or hTRPA1 with that caused by 1 mM menthol using a Ca^2+^-imaging method with Human embryonic kidney-derived 293 T (HEK293T) cells expressing hTRPM8 or hTRPA1. As expected, the effect of peppermint oil presented as the fura-2 ratio (corresponding to cytosolic Ca^2+^ concentrations) of changes by peppermint oil to those caused by menthol, the main component of peppermint oil, was nearly 1.0. Among the essential oils examined, clove oil and eucalyptus oil were found to exhibit some hTRPM8 activation (Figure 1A). Although many of the examined essential oils exhibited hTRPA1 activation like menthol, sage oil and eucalyptus oil showed less hTRPA1 activation (Figure 1B). When we calculated the Ratio of hTRPM8-activating ability versus hTRPA1-activating ability by simply dividing the values in Figure 1A by the values in Figure 1B, the Ratio of eucalyptus oil was comparable to that of peppermint oil and much higher than any other oil examined (Figure 1C).

### 1,8-cineole activates hTRPM8 but not hTRPA1

**Figure 2:**
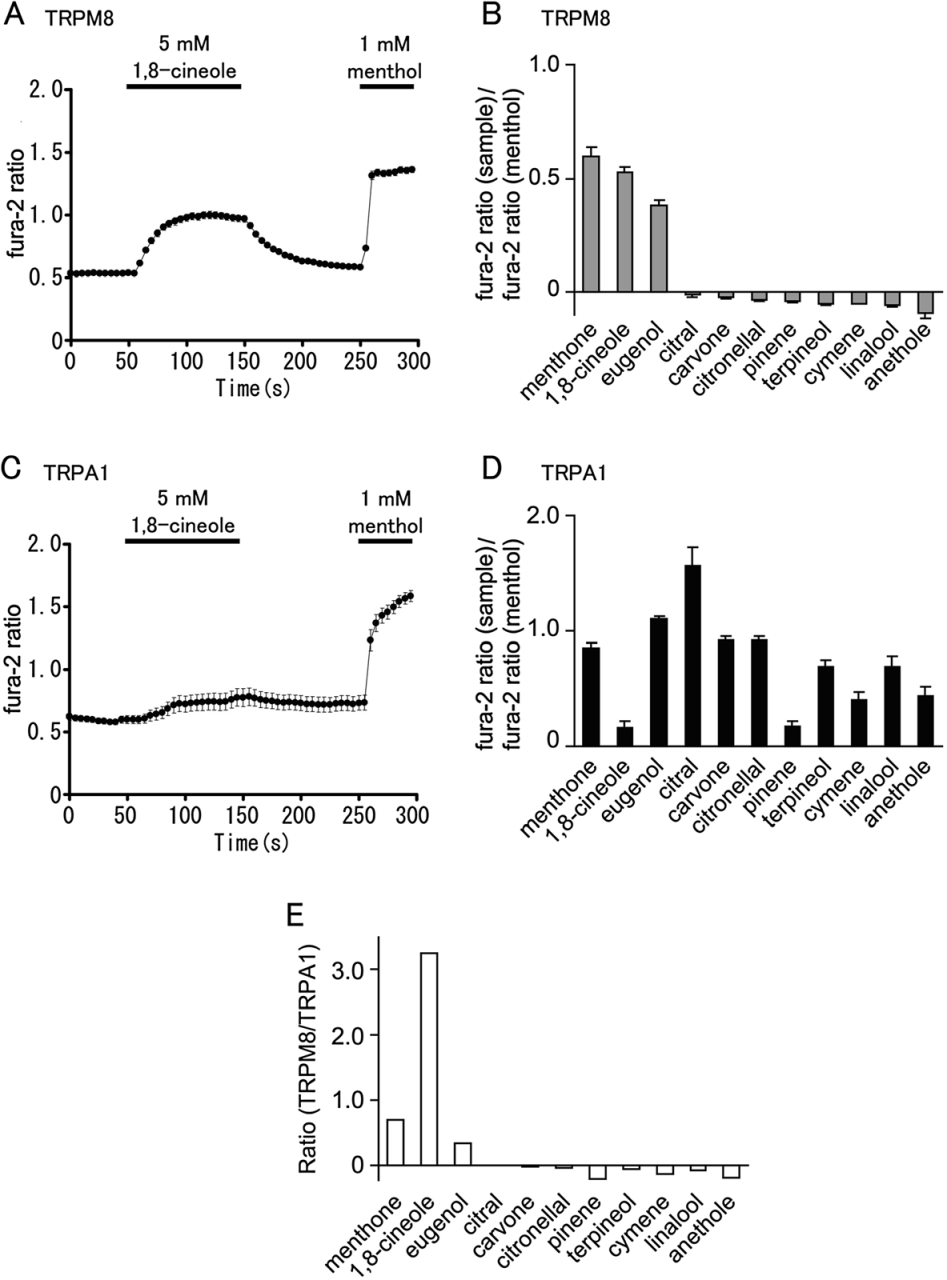
**Abilities of fragrance chemicals to activate hTRPM8 or hTRPA1. (A, C)** Effects of 1,8-cineole on fura-2 ratio in HEK293T cells expressing hTRPM8 (n = 32) (**A**) or hTRPA1 (n = 41) (**C**). **(B, D)** Comparison of the effects of fragrance chemicals (1 mM) on hTRPM8 (n = 16–80) (**B**) or hTRPA1 (n = 30–90) (**D**). Fura-2 ratio increases by each chemical were normalized to the fura-2 ratio increases by 1 mM menthol. **(E)** The Ratio of hTRPM8-activating ability versus hTRPA1-activating ability by dividing the values in (**B**) by the values in (**D**).

Next, we examined the effects of fragrance chemicals, many of which are contained in the essential oils tested above. We compared the same parameters used for the essential oils. As shown in Figure 2A and B, 1,8-cineole, menthone and eugenol showed relatively high response as measured by the sample/menthol fura-2 ratio in HEK293T cells expressing hTRPM8 compared with other chemicals. This is consistent with the observation that peppermint oil, clove oil and eucalyptus oil, which contain menthone, eugenol and 1,8-cineole, respectively, similarly showed relatively high ratios (Figure 1A). Interestingly, 1,8-cineole, but not menthone or eugenol, showed a low fura-2 ratio changes in HEK293T cells expressing hTRPA1 (Figure 2C, D). Although linalool was reported to produce antinociception [1], the Ratio of hTRPM8-activating ability versus hTRPA1-activating ability for linalool was found to be low, suggesting it was a poor candidate as an analgesic. Accordingly, the Ratio of 1,8-cineole was found to be very high as shown in Figure 2E. These data indicate that 1,8-cineole contained in eucalyptus oil can activate hTRPM8 without activating hTRPA1.

### 1,8-cineole acts on hTRPM8 and hTRPV3, but not on hTRPV1, hTRPV2 or hTRPA1

**Figure 3:**
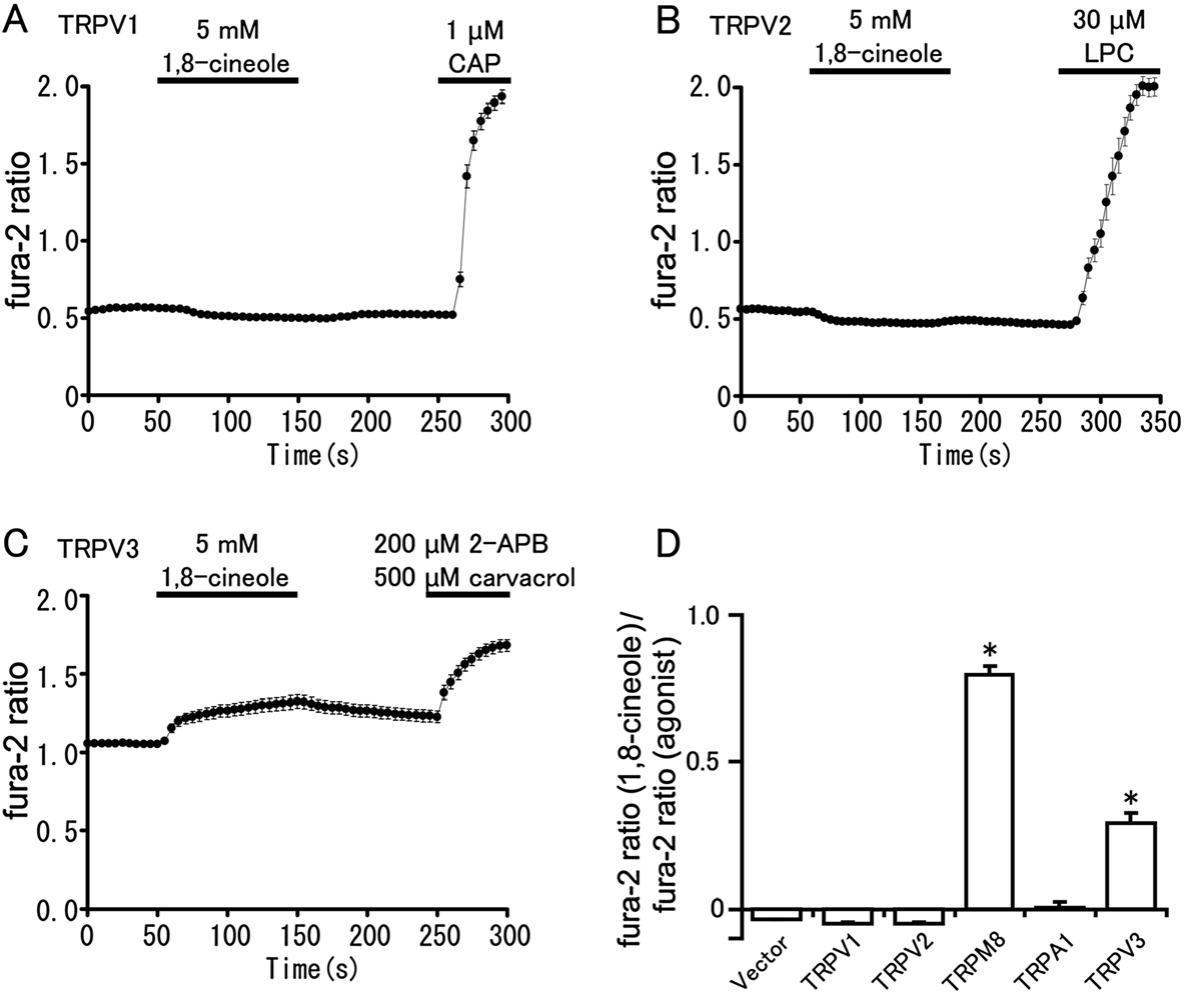
**Effects of 1,8-cineole on fura-2 ratio in HEK293T cells. (A-C)** Fura-2 ratio changes upon 1,8-cineole (5 mM) application in cells expressing hTRPV1 (n = 65) (**A**), hTRPV2 (n = 26) (**B**) or hTRPV3 (n = 79) (**C**). CAP (capsaicin), LPC (lysophosphatidylcholine), 2-APB (2-aminoethoxydiphenyl borate). Horizontal bars indicate duration of the applied stimuli. **(D)** 1,8-cineole caused significant fura-2 ratio increases in HEK293T cells expressing hTRPM8 (n = 32) or hTRPV3 (n = 79), but not in cells expressing hTRPV1 (n = 65), hTRPV2 (n = 26) or hTRPA1 (n = 41). Statistical significance was evaluated using ANOVA followed by two-tailed multiple t-test with Bonferroni correction. *: p < 0.05.

In order to examine whether 1,8-cineole can activate other TRP channels expressed in sensory neurons, we performed Ca^2+^-imaging experiments using HEK293T cells expressing hTRPV1 or hTRPV2 [6,24,32,33,35,40-44]. Treatment with 1,8-cineole increased the fura-2 ratio (340 nm/380 nm) in HEK293T cells expressing hTRPM8, but not in cells expressing hTRPA1, hTRPV1 or hTRPV2 (Figure 3A, B, D). These results are consistent with previous findings in rodent cell lines expressing these proteins. Because 1,8-cineole, like menthol, was reported to activate mouse TRPV3 in a *Xenopus* oocyte expression system [38,45,46], we checked the effect of 1,8-cineole on hTRPV3. Basal fura-2 ratio levels were slightly higher for hTRPV3-expressing HEK293T cells compared to cells expressing hTRPM8, hTRPA1, hTRPV1 or hTRPV2, probably because hTRPV3 can be activated by the warm temperatures to which the cells are exposed in the incubation conditions (Figure 3C). 1,8-cineole caused small but significant fura-2 ratio increase as expected (Figure 3C, D).

### 1,4-cineole activates hTRPM8 and hTRPA1 expressed in HEK293T cells

**Figure 4:**
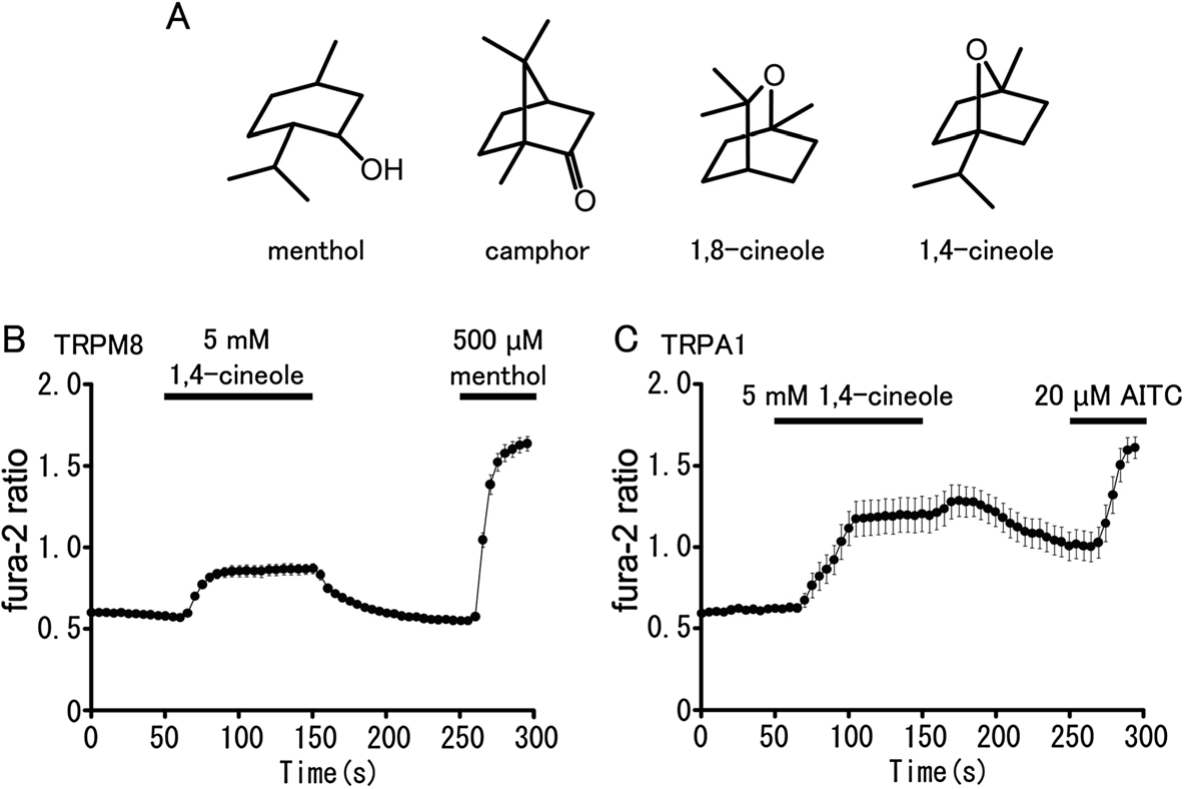
**1,4-cineole activates hTRPM8 and hTRPA1. (A)** Molecular structures of menthol, camphor, 1,8-cineole and 1,4-cineole. **(B)** 1,4-cineole increased fura-2 ratio (340/380 nm) in cells expressing hTRPM8 (n = 35). **(C)** 1,4-cineole increased fura-2 ratio in cells expressing hTRPA1 (n = 17).

Since eucalyptus oil contains not only 1,8-cineole but also 1,4-cineole, and because these chemicals have similar structures (Figure 4A), the actions of 1,4-cineole on hTRPM8 and hTRPA1 were assessed using a Ca^2+^-imaging method. Surprisingly, 1,4-cineole (5 mM) caused fura-2 ratio increases not only in cells expressing hTRPM8 (Figure 4B), but also in cells expressing hTRPA1 (Figure 4C). This result might explain the apparent difference in the effects between eucalyptus oil and 1,8-cineole regarding the ratio of hTRPM8-activating versus hTRPA1-activating abilities (Figure 1C, 2E) in which the ratio for 1.8-cineole is very low while the ratio for eucalyptus oil is comparable to that of peppermint oil.

### 1,4-cineole but not 1,8-cineole activates hTRPA1

**Figure 5:**
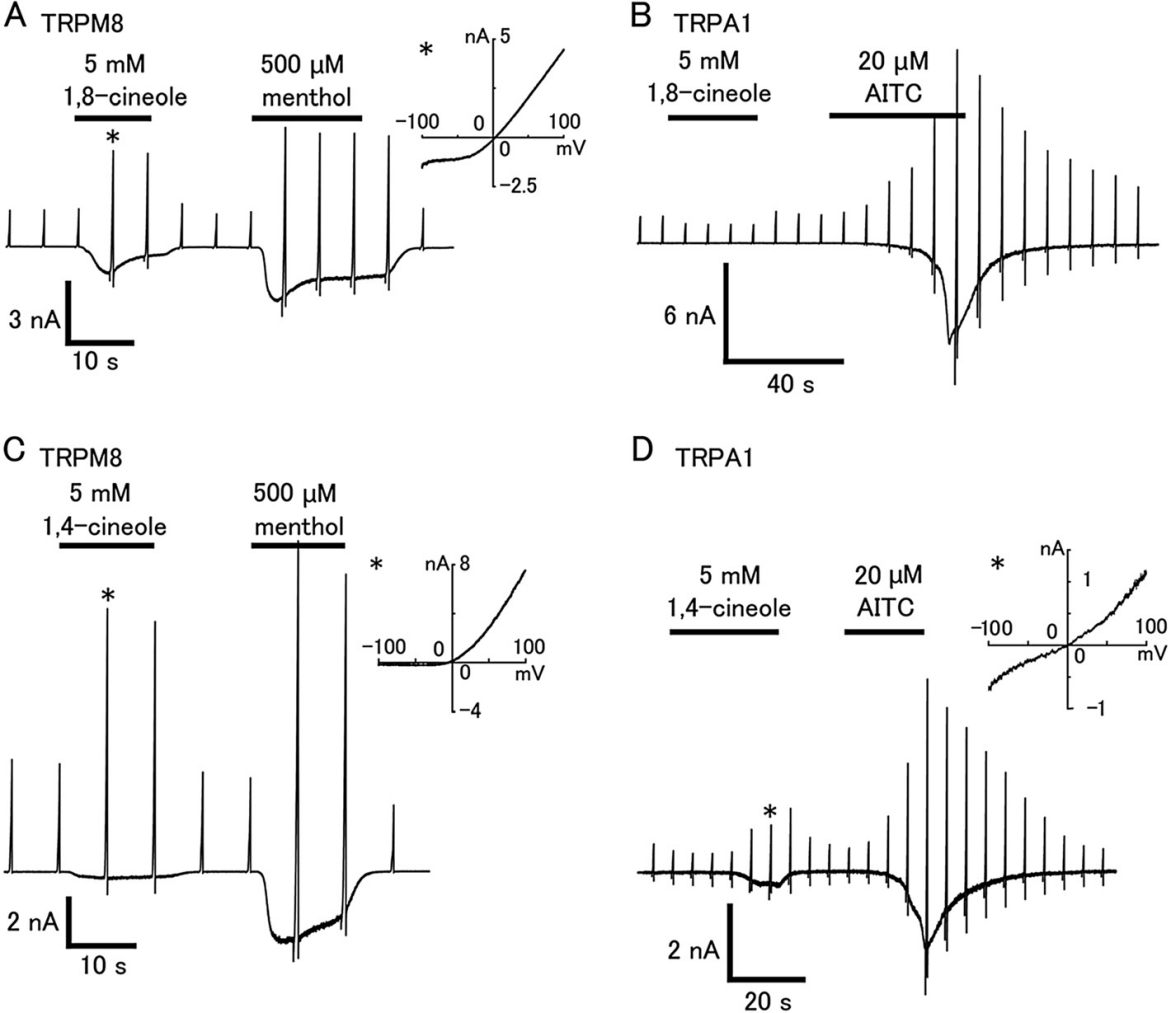
**Effects of 1,8- or 1,4-cineole on HEK293T cells expressing hTRPM8 or hTRPA1. (A, B)** 1,8-cineole (5 mM) activated hTRPM8 (**A**) with an outwardly rectifying current–voltage relationship, but not hTRPA1 (B). **(C, D)** 1,4-cineole activated both hTRPM8 and hTRPA1 with an outwardly rectifying current–voltage relationship. The insets (**A, C **and **D**) indicate the current–voltage relationship at the point indicated by * in the left trace.

Next, we performed patch-clamp experiments to confirm the effects of 1,8-cineole and 1,4-cineole on hTRPM8 and hTRPA1 expressed in HEK293T cells. Both 1,8- and 1,4-cineole (5 mM) evoked inward currents with outwardly rectifying current–voltage (I-V) relationship in cells expressing hTRPM8 (Figure 5A, C). On the other hand, 1,8-cineole (5 mM) did not activate hTRPA1 in cells responding to AITC, a TRPA1 agonist, while 1,4-cineole evoked an inward current with outwardly rectifying I-V relationship in cells expressing hTRPA1 (Figure 5B, D), showing that similar structural chemicals exhibited different effects on hTRPA1. Menthol has bimodal action on mTRPA1; lower concentrations of menthol activate mTRPA1 whereas higher concentrations of menthol inhibit it [22]. To confirm whether low concentrations of 1,8-cineole activate hTRPA1, we performed patch-clamp experiments. 10 μM and 100 μM 1,8-cineole did not activate hTRPA1 in cells responding to AITC (data not shown).

### 1,8-cineole inhibits hTRPA1 currents activated by different agonists

**Figure 6:**
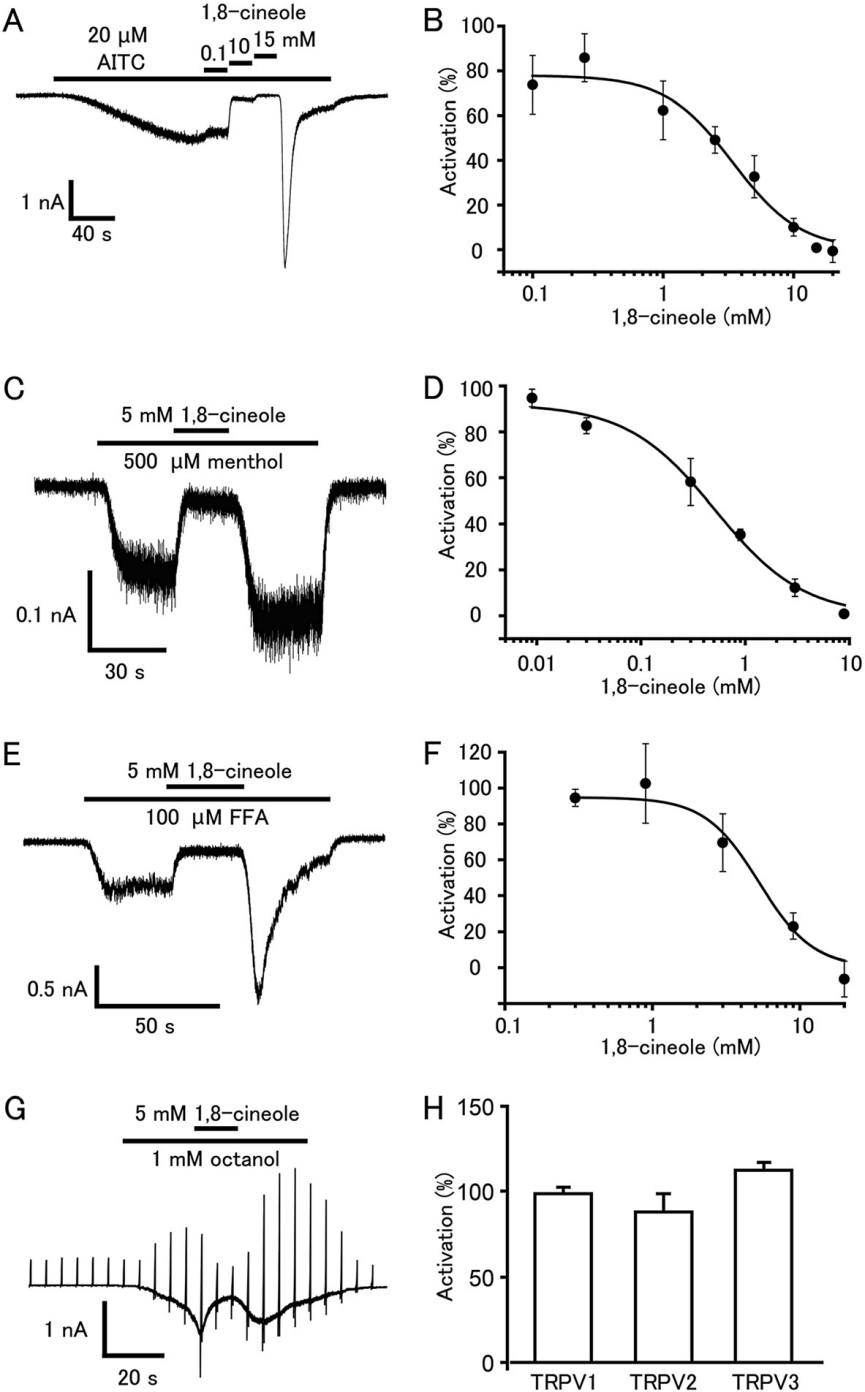
**1,8-cineole inhibits but does not activate hTRPA1-mediated currents in HEK293T cells. (A)** A representative AITC (20 μM)-evoked hTRPA1 current that was inhibited by 1,8-cineole in a dose-dependent manner in the absence of extracellular Ca^2+^. **(B)** Dose-dependent inhibition of AITC (20 μM)-evoked hTRPA1 current by 1,8-cineole. IC_50_ and Hill’s coefficient values are 3.4 ± 0.6 mM and 1.7 ± 0.4, respectively. Data are shown as the mean ± SEM (n = 5–8). **(C)** A representative whole-cell menthol (500 μM)-evoked hTRPA1 current that was inhibited by 1,8-cineole (5 mM) in the absence of extracellular Ca^2+^. **(D)** Dose-dependent inhibition of menthol (500 μM)-evoked hTRPA1 current by 1,8-cineole. IC_50_ and Hill’s coefficient values are 0.5 ± 0.1 mM and 1.0 ± 0.2, respectively. Data are shown as the mean ± SEM (n = 5–8). **(E)** A representative whole-cell FFA (100 μM)-evoked hTRPA1 current that was inhibited by 1,8-cineole (5 mM) in the absence of extracellular Ca^2+^. **(F)** Dose-dependent inhibition of FFA (100 μM)-evoked hTRPA1 current by 1,8-cineole. IC_50_ and Hill’s coefficient values are 5.3 ± 0.1 mM and 2.4 ± 0.8, respectively. Data are shown as the mean ± SEM (n = 6–8). **(G)** A representative whole-cell octanol (1 mM)-evoked hTRPA1 current that was inhibited by 1,8-cineole (5 mM) in the presence of extracellular Ca^2+^. **(H)** 1,8-cineole did not inhibit hTRPV1 (n = 25), hTRPV2 (n = 50) or hTRPV3 (n = 32 responses) by capsaicin, 2-APB or cocktail (2-APB + carvacrol), respectively.

Because 1,8-cineole has analgesic and anti-inflammatory effects *in vivo*[47,48], we hypothesized that 1,8-cineole inhibits TRPA1 [24]. hTRPA1 currents induced by AITC (20 μM), a TRPA1 agonist that acts through cysteine covalent modification [19,20], were inhibited by 1,8-cineole in a dose-dependent manner with a half-maximal inhibition (IC_50_) of 3.4 ± 0.6 mM (Figure 6A, B). The effects of 1,8-cineole on hTRPA1 activated by other TRPA1 agonists were also determined. Several TRPA1 agonists with different activation mechanisms were chosen: menthol, which seems to interact specifically with residues within transmembrane domain 5 to gate TRPA1 [22]; flufenamic acid (FFA), thought to be a cysteine-nonreactive compound [21]; and octanol, whose action is largely unknown [49]. For menthol- or FFA-evoked hTRPA1 currents, we measured the current responses in the absence of extracellular Ca^2+^ to minimize desensitization similarly to the experiment examining the effects on AITC-evoked currents. For examination of octanol-evoked hTRPA1 currents, we collected patch-clamp recordings in the presence of extracellular Ca^2+^ because octanol-evoked responses were too small to analyze in the absence of extracellular Ca^2+^ (data not shown), thus leading to difficulties in plotting a dose-dependent curve. Similar to hTRPA1 currents activated by AITC, hTRPA1 currents activated by menthol (500 μM) or FFA (100 μM) were inhibited by 1,8-cineole in a dose-dependent manner with an IC_50_ value of approximately 0.5 ± 0.1 or 5.3 ± 0.1 mM, respectively (Figure 6C, D, E, F). Octanol (1 mM)-evoked hTRPA1 currents were inhibited reversibly by 1,8-cineole (5 mM) (Figure 6G). These results again suggest that 1,8-cineole is an antagonist of hTRPA1. Interestingly, AITC-, menthol-, or FFA-evoked currents were increased upon washout of 1,8-cineole (Figure 6A, C, E) probably through the release of blocking by 1,8-cineole. We confirmed that 1,8-cineole did not inhibit hTRPV1, hTRPV2 and hTRPV3 responses activated by capsaicin, 2-APB and cocktail (2-APB + carvacrol), respectively (Figure 6H).

### 1,8-cineole inhibits mouse DRG neuron activated by TRPA1 agonist

**Figure 7:**
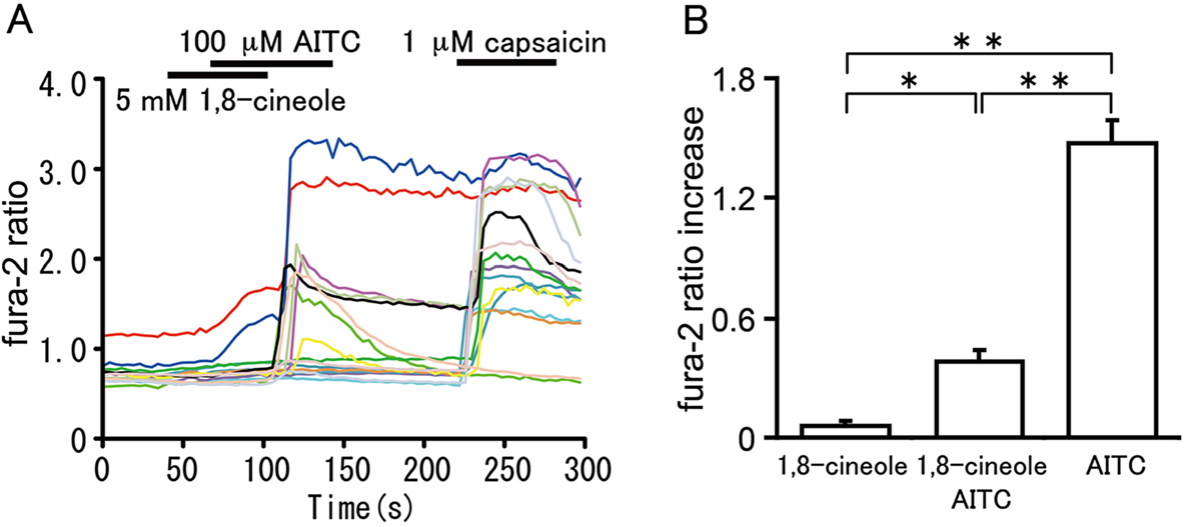
**Inhibitory effect of 1,8-cineole in mouse DRG neurons. (A)** Inhibitory effects of 1,8-cineole (5 mM) on fura-2 ratio (340/380 nm) increases caused by 100 μM AITC (n = 42). **(B)** 1,8-cineole can significantly inhibit fura-2 ratio increases caused by AITC (100 μM) in mouse DRG neurons ( n = 42). Statistical significance was made by using ANOVA with the Bonferroni’s post-hoc test. *: p < 0.05 **: p < 0.01.

To confirm the inhibitory effect of 1,8-cineole on TRPA1 in native sensory neurons, we performed Ca^2+^-imaging experiments using isolated mouse dorsal root ganglion (DRG) neurons. We examined the ability of 1,8-cineole to inhibit fura-2 ratio increases by AITC. Almost all AITC-sensitive neurons were inhibited by 1,8-cineole (5 mM) and the inhibition was statistically significant (Figure 7).

### 1,8-cineole inhibits sensory irritation caused by hTRPA1 agonists in vivo

**Figure 8:**
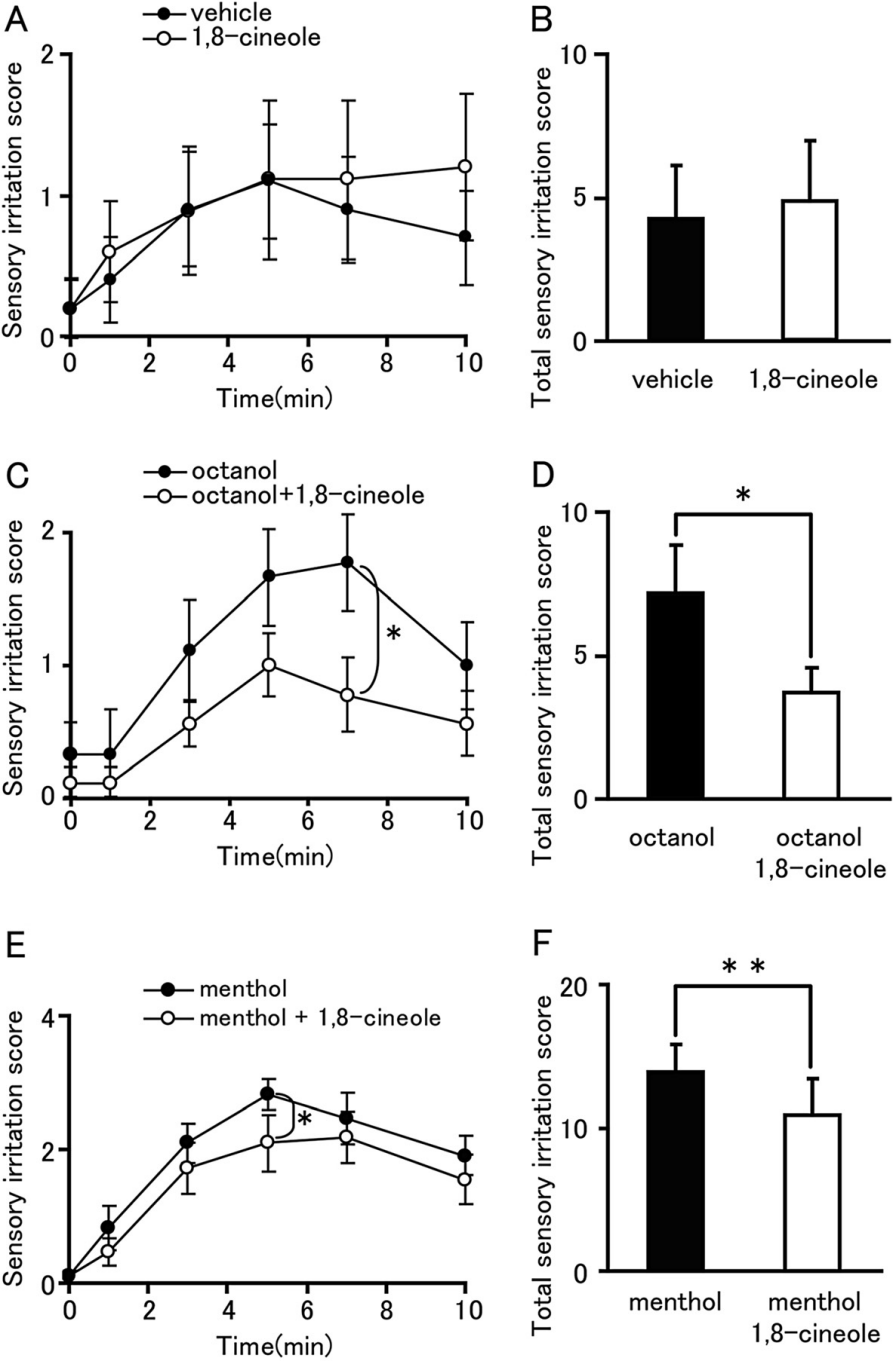
**Inhibitory effect of 1,8-cineole in sensory irritation tests in humans.** (**A** and **B**) 0.5 (wt%) 1,8-cineole did not cause a difference of sensory irritation scores compared with vehicle (n = 10). **(C)** Sensory irritation caused by 0.2 (wt%) octanol was significantly inhibited by concomitant application of 0.1 (wt%) 1,8-cineole 7 min after application. **(D)** Total score of sensory irritation by octanol was significantly inhibited by 1,8-cineole. Statistical significance was evaluated using Wilcoxon signed-rank test. *: p < 0.05. n = 11. **(E)** Sensory irritation caused by 0.5 (wt%) menthol was significantly inhibited by concomitant application of 0.5 (wt%) 1,8-cineole 5 min after application. **(F)** Total score of sensory irritation by menthol was significantly inhibited by 1,8-cineole. Statistical significance was evaluated using Wilcoxon signed-rank test. *: p < 0.05. n = 11.

To confirm the inhibitory effect of 1,8-cineole on hTRPA1 *in vivo*, sensitive human subjects were recruited for sensory irritation tests. Sensory irritation caused by 1,8-cineole itself was comparable to that by vehicle alone (Figure 8A, B), indicating that 1,8-cineole does not cause sensory irritation. Next, we examined inhibitory effect of 1,8-cineole with concomitant application of the TRPA1 agonist octanol [49] and 1,8-cineole. Octanol (0.2 wt%) caused sensory irritation for 7 min with a gradual increase after application (Figure 8C), as was previously reported [50]. The octanol-induced sensory irritation was significantly reduced by concomitant application of 1,8-cineole at the 7 min time point (Figure 8C). Analysis of the total sensory irritation score indicated that 1,8-cineole significantly inhibited sensory irritation caused by octanol (Figure 8D). This further supports the inhibitory action of 1,8-cineole on octanol-induced irritation, which could involve regulation of TRPA1 activity. Menthol is known to activate both TRPM8 and TRPA1 which can simultaneously cause comforting and irritating sensations. In order to examine whether 1,8-cineole can reduce the menthol-induced irritation, we applied menthol with or without 1,8-cineole. Concomitant application of menthol with 1,8-cineole significantly reduced irritation probably through inhibition of TRPA1 by 1,8-cineole (Figure 8E, F).

## Discussion

In this study, we screened several essential oils and fragrance chemicals to find substances that activate hTRPM8 but not hTRPA1, which presumably cause a comforting sensation. We identified 1,8-cineole as a substance with these properties although we used one concentration of 1,8-cineole (5 mM). Furthermore, 1,8-cineole could inhibit hTRPA1, suggesting this substance might act as an analgesic.

TRPA1 is an excitatory ion channel targeted by pungent irritants such as those from mustard oil and garlic and is thought to function in diverse sensory processes, including cold nociception and inflammatory pain. Therefore, TRPA1 is considered to be a promising target for use in identifying analgesic drugs. A natural analgesic compound that does not accelerate pain signaling is desirable for pharmaceutical or cosmetic pain relief. Several reports showed that TRPA1 antagonists, such as ruthenium red, HC-030031, AMG5445, A967079 and camphor, possess analgesic properties [26-31]. Of these, camphor is the only naturally occurring compound and is often used in cosmetics because of its minimal adverse effects. However, camphor is not suited for use as an analgesic compound because it causes a warm and hot sensation [39]. It has become clear that this warm and hot sensation is mediated through activation of TRPV1 [31,38]. Moreover, TRPM8 contributes to sensing unpleasant cold stimuli or mediating the effects of cold analgesia [34,35]. Although menthol, the main ingredient of peppermint, is used for pain relief in daily life through TRPM8 activation [35], its ability to activate hTRPA1 restricts widespread use of menthol as an analgesic [36]. Therefore, chemicals that activate TRPM8 and inhibit TRPA1, but do not activate TRPV1, would be ideal as analgesic agents.

We found that activation of hTRPA1 induced by several agonists with different activation mechanisms can be inhibited by 1,8-cineole. Moreover, 1,8-cineole activated hTRPM8 and hTRPV3, but not hTRPA1, hTRPV1 or hTRPV2. It was recently shown that both peripheral and central activation of TRPM8 could produce an analgesic effect that specifically reverses the sensitization of behavioral reflexes elicited by peripheral nerve injury [34-36]. From this point of view, 1,8-cineole appears to be an ideal natural analgesic that activates hTRPM8 and inhibits hTRPA1.

1,8-cineole is known to act as an agonist of the TRPM8 channel with lower efficacy and potency (3.4 ± 0.4 mM) on TRPM8 than menthol [32,38]. 1,8-cineole also activates the TRPV3 channel in mice, but not the western clawed frog TRPV3 [45]. Furthermore, 1,8-cineole inhibits the chemical nociception produced by several irritants, and has an anti-inflammatory efficacy in patients with severe asthma [51]. The present study suggests that the known analgesic and anti-inflammatory actions of 1,8-cineole can be attributed to its TRPM8-activating and TRPA1-inhibiting abilities.

1,8-cineole has a fresh smell and elicits a cooling sensation when ingested or applied to the skin and is a common additive in flavorings, food, mouthwashes and cough suppressants. 1,8-cineole is also often used in aromatherapy, as a stimulant in skin baths, by the pharmaceutical industry in drug formulations to enhance percutaneous penetration and as a decongestant and antitussive [52-54]. Experimental data have shown that 1,8-cineole is an analgesic and anti-inflammatory agent with beneficial effects for patients with severe asthma [51]. Although inhibitory effects of 1,8-cineole on the formation of prostaglandins and cytokines by stimulated monocytes have been observed *in vitro*, the molecular targets and mechanisms of the analgesic effect of 1,8-cineole remain unclear [55].

In a human study, we examined whether 1,8-cineole could inhibit sensory irritation caused by octanol and menthol with senseitive volunteers. Because of the clinical setting, especially in the cosmetic research field, both menthol and octanol are well-known chemicals causing skin irritation, and neither cinnnamaldehyde nor allicin is used for human skin studies. The result that 1,8-cineole, whose ability to activate TRPM8 is lower than menthol, inhibited menthol-evoked skin irritation clearly suggests that the inhibitory effects of 1,8-cineole are probably due to inhibition of TRPA1 but not activation of TRPM8.

The inhibitory effects of 1,8-cineole on menthol-induced hTRPA1 activation was a little greater than those for AITC- or FFA-induced hTRPA1 activation (Figure 6B, D, F). Menthol has bimodal action through transmembrane domain 5 of TRPA1 in some species [22]. Therefore, the similarity between the molecular structures of menthol and 1,8-cineole (Figure 4A) suggests that 1,8-cineole could act on the same domain of TRPA1 as menthol, although the structural basis for menthol-evoked hTRPA1 activation is not known. Four compounds with similar structures (Figure 4A) exhibited different effects on hTRPM8 and hTRPA1: i) menthol and 1,4-cineole activate both hTRPM8 and hTRPA1 [56]; ii) camphor inhibits hTRPA1 [31]; iii) 1,8-cineole activates hTRPM8 and inhibits hTRPA1 (Figures 2, 3, 4). The fact that the four compounds exhibit promiscuous effects on hTRPM8 and hTRPA1 suggests that more detailed analyses would lead to a better understanding of the structural basis for the action of these compounds on TRPM8 and TRPA1.

## Conclusions

1,8-cineole was found to be a rare natural antagonist of hTRPA1. 1,8-cineole activates hTRPM8 but inhibits hTRPA1 activated by several agonists. Moreover, the sensory irritation caused by octanol or menthol, TRPA1 agonists, was inhibited by concomitant 1,8-cineole application in humans. Thus, the analgesic and anti-inflammatory effects of 1,8-cineole might be related to its capacity to inhibit TRPA1 activity, suggesting there may be many effective uses for 1,8-cineole based on its unique action on TRPM8 and TRPA1.

## Methods

### Molecular cloning

Full-length hTRPA1, hTRPM8, hTRPV1, and hTRPV2 were obtained from Life Technologies (Carlsbad, CA, USA) and hTRPV3 was generously provided by Dr. Hwang (Korea University). cDNAs were cloned into the pcDNA3.1 vector.

### Cell culture

HEK293T cells were maintained in DMEM (WAKO Pure Chemical Industries, Ltd., Osaka, Japan) supplemented with 10% FBS (Biowest SAS, Caille, France), 100 units/mL penicillin (Life Technologies Corp., Carlsbad, CA, USA), 100 μg/mL streptomycin (Life Technologies Corp.), and 2 mM L-glutamine (GlutaMAX, Life Technologies Corp.) at 37°C in 5% CO_2_. For Ca^2+^-imaging, 1 μg plasmid DNA containing hTRPA1, hTRPV1, hTRPV2, hTRPV3 or hTRPM8 in pcDNA3 in OPTI-MEM medium (Life Technologies Corp.) were transfected into HEK293T cells using Lipofectamine Plus Reagent (Life Technologies Corp.). After incubating for 3 to 4 h, the cells were reseeded on coverslips and further incubated at 37°C in 5% CO_2_.

### Animals

Male C57BL/6 mice (4–5 weeks, SLC, Shizuoka, Japan) were used. Animals were housed in a controlled environment (12 h light/dark cycle, room temperature 22–24°C, 50–60% relative humidity) with free access to food and water. All procedures involving the care and use of animals were approved by The Institutional Animal Care and Use Committee of National Institutes of Natural Sciences and performed in accordance with the *Guide for the Care and Use of Laboratory Animals* (National Institutes of Health publication number 85–23, revised 1985).

### Preparation of primary mouse DRG neurons

Mouse dorsal root ganglions (DRGs) were dissected from mice, incubated with 1.25 mg/mL collagenase (Sigma-Aldrich) at 37°C for 15 min, and dissociated using mechanical trituration. After filtration with a cell strainer (70 μm, BD, Franklin Lakes, USA), cells were plated on poly-D-lysine-coated coverslips and incubated in medium (MEM supplemented with 10% FBS, penicillin, streptomycin, and l-glutamine) containing nerve growth factor (100 ng/mL).

### Human subjects

Japanese male subjects in their 20s and 30s were selected as participants to eliminate confounding factors that may influence the perception of sensitive skin, including race, age, gender, and hormonal and psychosocial interactions. To evaluate sensory irritation, we selected skin sensitive male volunteers. Female volunteers were excluded because of possible hormonal influences. Ethics approval and informed consent was obtained from all participants.

### Ca^2+^-imaging

Ca^2+^-imaging was performed 1 day after transfection. HEK293T cells on coverslips were mounted in an open chamber and superfused with standard bath solution (140 mM NaCl, 5 mM KCl, 2 mM MgCl_2_, 2 mM CaCl_2_, 10 mM HEPES, 10 mM glucose, pH 7.4). Cytosolic-free Ca^2+^ concentrations in HEK293T cells were measured by dual-wavelength fura-2 (Molecular Probes, Invitrogen Corp.) microfluorometry with excitation at 340/380 nm and emission at 510 nm. The fura-2 ratio image was calculated and acquired using the IP-Lab imaging processing system (Scanalytics Inc., Fairfax, VA USA).

### Electrophysiology

Whole-cell patch-clamp recordings were performed 1 day after transfection. The standard bath solution was the same as that used in the Ca^2+^-imaging experiments, and extracellular Ca^2+^ was removed and 5 mM EGTA was added for the recording of AITC-, menthol- and FFA-induced current responses. The pipette solution contained 140 mM KCl, 5 mM EGTA, 10 mM HEPES, pH 7.4 (adjusted with KOH). Data from whole-cell voltage-clamp recordings were sampled at 10 kHz and filtered at 5 kHz for analysis (Axon 200B amplifier with pCLAMP software, Axon Instruments, Sunnyvale, CA, USA). Membrane potential was clamped at −60 mV and voltage ramp-pulses from −100 to +100 mV (500 ms) were applied every 5 sec. All experiments were performed at room temperature.

### Sensory irritation tests

The study was conducted at a temperature of 21–23°C and a relative humidity of 45-55%. Areas of skin were cleaned with a wet towel and acclimatized for 10 min prior to testing. Blind randomized half-region (left vs. right) trials were performed with two different samples applied to the neck region. A total of 200 μl of base was applied. The subjects evaluated pricking, stinging, burning and itching sensations after 1, 3, 5, 7 and 10 min of compound/chemical application in accordance with the criteria summarized in Table 1. The total sensory irritation scores were calculated for the entire period.

**Table 1:** Sensory irritation scores

**Sensory perception**	**Score**	**Scoring criteria**
	5	Unbearable intense sensation
Itching	4	
Slightly unusual	3	Distinct sensation
Stinging pain	2	
Burning sensation	1	Obscure sensation

### Data analysis

Data in all figures are shown as means ± standard error of the mean (SEM). Statistical significance of effects of 1,8-cineole on several TRP channels were evaluated using ANOVA followed by two-tailed multiple t-test with Bonferroni correction, inhibitory effects of 1,8-cineole on mouse DRG neuron activation were made by using ANOVA with the Bonferroni’s post-hoc test. Sensory irritation tests were evaluated using a Wilcoxon signed-rank test.

## Abbreviations

AITC: Allyl isothiocyanate; FFA: Flufenamic acid; HEK: Human embryonic kidney; TRPA1: Transient receptor potential ankyrin 1; TRPM8: Transient receptor potential melastatin 8; TRPV: Transient receptor potential vanilloid; CAP: Capsaicin; LPC: Lysophosphatidylcholine; 2-APB: 2-aminoethoxydiphenyl borate.

## Competing interests

The authors have no financial or other relationship that could lead to a conflict of interest.

## Authors' contributions

MT, FF, KU and MT designed the experiments, and MT, FF, KU performed the in vitro experiments and analysis. SY, MS, CH and MS performed the human study. MT, FF, KU and MT prepared the manuscript. All authors read and approved the final manuscript.
